# Racial differences between African-American and white women in insulin resistance and visceral adiposity are associated with differences in apoCIII containing apoAI and apoB lipoproteins

**DOI:** 10.1186/1743-7075-11-56

**Published:** 2014-12-17

**Authors:** Liyun Wang, Frank M Sacks, Jeremy D Furtado, Madia Ricks, Amber B Courville, Anne E Sumner

**Affiliations:** Department of Nutrition, Harvard School of Public Health, 665 Huntington Avenue, Building 1, Room 201, Boston, MA 02115 USA; Channing Division of Network Medicine, Department of Medicine, Brigham and Women’s Hospital and Harvard Medical School, Boston, MA USA; Diabetes, Endocrinology and Obesity Branch, National Institute of Diabetes, Digestive and Kidney Diseases, National Institutes of Health, Bethesda, MD USA; Nutrition Department, Clinical Center, National Institutes of Health, Bethesda, Maryland

**Keywords:** ApoAI, ApoCIII, HDL, ApoB lipoproteins, Visceral adipose tissue, Insulin resistance, African-Americans, Coronary heart disease

## Abstract

**Background:**

African-Americans have higher HDL, less visceral adipose tissue (VAT) and lower triglyceride (TG) and apoCIII concentrations than whites, despite being more insulin-resistant. We studied in African-American and white women the influences of insulin resistance and VAT on the apoAI concentrations of two HDL subspecies, one that contains apoCIII that is associated with increased risk of coronary heart disease (CHD) and one that does not have apoCIII that is associated with decreased CHD; and on the apoCIII concentrations of HDL and of the apoB lipoproteins.

**Methods:**

The participants were 32 women (14 African-Americans, 18 white) of similar age (39 ± 12 vs. 42 ± 11y). Mean BMI was 34 kg/m^2^ in the African-Americans compared to 30 in the whites. A standard diet (33% fat, 52% carbohydrate, 15% protein) was provided for 7 days followed by a test meal (40% fat, 40% carbohydrate, 20% protein) on Day 8. Insulin sensitivity index (S_I_) was calculated from the minimal model.

**Results:**

After controlling for S_I_, African-Americans have a higher mean apoAI level in HDL with apoCIII compared with whites (12.9 ± 2.8 and 10.9 ± 2.9 mg/dL, respectively, *P* = 0.05). S_I_ was associated with higher apoAI in HDL with apoCIII, whereas VAT was not associated with this HDL subspecies. This pattern of results was reversed for apoCIII concentrations in apoB lipoproteins. After adjusting for S_I_, African-Americans had lower apoCIII in apoB lipoproteins. S_I_ was associated with lower apoCIII in total apoB lipoproteins, whereas VAT was associated with higher apoCIII in all the apoB lipoproteins. Additional adjustment for VAT tended to reduce the difference in apoCIII between the groups.

**Conclusions:**

African-American women have a higher HDL with apoCIII level than whites when controlled for insulin sensitivity. African-Americans have lower insulin sensitivity. Insulin sensitivity is associated with higher levels of HDL with apoCIII. ApoCIII levels in VLDL are lower in African-American women than whites, also affected by insulin sensitivity which is associated with low apoCIII in VLDL. VAT has a strong association with apoCIII in apoB lipoproteins but not with apoAI in HDL with apoCIII.

**Trial registration:**

ClinicalTrials.gov Identifier: NCT00484861

## Background

According to the US National Health and Nutrition Examination Survey (NHANES III), African-Americans have higher prevalence of cardiovascular disease compared with whites [1, 2]. However, the metabolic basis for this is unknown. Paradoxically, African-Americans have less dyslipidemia compared to whites; HDL-cholesterol (HDL-C) is higher and triglycerides (TG) is lower [3–5]. Other factors complicating our understanding of the causes of CHD in African-Americans are insulin resistance, which is higher, and visceral adipose tissue (VAT) which is lower in African-Americans than whites [4, 6, 7]. Insulin resistance is a predictor of CHD risk and it is correlated with dyslipidemia [8]. If insulin resistance is a causal factor in dyslipidemia, then African-Americans would be expected to have more dyslipidemia than whites; however the opposite is true. Visceral adipose tissue (VAT) is correlated with dyslipidemia, and the lower mass of VAT in African-Americans is consistent with less dyslipidemia. However, VAT is generally correlated with insulin resistance in African-American and white populations [9, 10]. Thus, it is unknown why TG and VAT are not usually elevated in insulin-resistant African-American women. It is possible that the usual grouping of risk factors and metabolic influences, dyslipidemia, insulin resistance, and visceral fat, recognized in the concept of the metabolic syndrome and strong predictors of CHD, may not apply as well to African-Americans as whites. A clinical implication of this paradox is that CHD screening panels that involve HDL-c and TG may not detect risk as well in African-Americans [11–13]. That could be true if the favorable HDL and TG levels in African-Americans mask unfavorable lipoprotein subspecies and apolipoproteins that are related to high CHD risk.

ApoCIII is an important predictor of CHD risk [14, 15]. In the circulation, apoCIII is associated with apoB lipoproteins and enhances the atherogenicity of very low density lipoproteins (VLDL) and low density lipoproteins (LDL) [14, 16]. Evidence is accumulating that apoCIII concentrations of apoB containing lipoproteins are strong predictors of CHD [14, 16–18]. ApoCIII concentration in VLDL + LDL is a more specific predictor of CHD risk than plasma TG [16]. Furthermore, we recently reported, after dividing apoB containing lipoproteins to VLDL and LDL subfractions, apoCIII levels in VLDL and LDL were each associated with CHD [14]. Several studies show that African-Americans have lower apoCIII level than whites [19, 20], and we previously reported that the low apoCIII level contributes to the low TG concentrations in African-Americans [21].

HDL-C subtypes based on content of apoCIII are differentially associated with risk of CHD. HDL without apoCIII, the major HDL type, is inversely associated with CHD. However, a minor subclass, accounting for around 13% of HDL cholesterol, carries apoCIII, and is linked to a higher risk of CHD. Participants with plasma HDL apoCIII concentrations in the highest compared to the lowest 20% of the population had a 60% increased risk of CHD [15]. Although African-Americans have higher HDL-C and apoAI levels than whites [3, 19], it is unknown if HDL with apoCIII or HDL without apoCIII is different by race.

Most studies of lipids and cardiovascular risk have used fasting concentrations. However, recent studies have suggested that the postprandial TG level is a better predictor than the fasting TG level for assessing present and future CVD events in the nondiabetic population [22, 23]. There is little known about postprandial lipids and apolipoproteins in African-Americans compared to whites.

Previously we reported the determinants of triglyceride levels in the participants in this research study [21]. We found that low concentrations of apoCIII and VAT contributed to the low triglyceride concentrations in African-Americans. In the present study, we extended this approach to explore race differences in potentially dysfunctional HDL subspecies that we newly identified [15]. In addition, increasing understanding of apoCIII as a potentially causal risk factor [24], in its own right, led us to explore determinants of apoCIII concentrations in HDL and apoB lipoproteins. We focused on the postprandial period, hypothesizing that the postprandial lipoproteins can give us more information on race differences in HDL subspecies and apoCIII contents of HDL and apoB lipoproteins. We assessed insulin sensitivity by a frequently sampled intravenous glucose tolerance test because we anticipated that insulin sensitivity could affect the apoC-III containing lipoproteins [25, 26]. Our goal was first to determine whether postprandial apoAI in HDL with or without apoCIII and apoCIII in apoB lipoproteins differ between African-Americans and whites, and second to determine if differences in insulin resistance or VAT influence the racial differences between groups in the lipoprotein subspecies. Indeed we found that lower insulin sensitivity in African-Americans compared to whites obscured differences in apoCIII containing lipoproteins between the racial groups.

## Methods

### Subjects

Nondiabetic African-American and white women of similar age and BMI were enrolled to minimize the confounding effects of age and obesity. Recruitment was by flyers, newspaper advertisements and website. Exclusion criteria were use of medications, vitamins or food supplements which affect either glucose or lipid metabolism; anemia, liver, kidney or thyroid dysfunction. The final study group had 32 non-diabetic women (14 African-Americans and 18 whites). Age and BMI range are 22 to 59 years and 20.6 to 45.9 kg/m^2^, respectively. Most of the subjects were part of a previous report on determinants of triglyceride levels [21]. The study was approved by the Institutional Review Board of the National Institute of Diabetes, Digestive and Kidney Diseases. All participants gave written informed consent.

### Protocol

At the screening visit, a medical history, physical examination, measurement of hematology and blood chemistry, and an EKG were performed. The methods for measuring insulin resistance and body composition and the study protocol in detail have been reported previously [21]. Briefly, at the second visit an insulin-modified frequently sampled intravenous glucose tolerance test (IM-FSIGT) was performed. After a 12 hours overnight fast each participant came to the Clinical Center at 7 AM. Intravenous catheters were placed in each antecubital vein. Dextrose (0.3 g/kg) was administered intravenously over 1 minute. An insulin (0.03 U/kg) bolus was injected at 20 min. Blood samples were taken at -10, -1, 0, 1, 2, 3, 4, 5, 6, 7, 8, 10, 12, 14, 16, 19, 22, 23, 24, 25, 27, 30, 40, 50, 60, 70, 80, 90, 100, 120, 150, 180 min. Glucose and insulin concentrations were entered into the minimal model and the insulin sensitivity index (S_I_) (MinMOD Millenium v.6.02) calculated. The insulin sensitivity index is a calculated measure of whole body insulin sensitivity. In the absence of diabetes, the more resistance there is to insulin’s ability to promote glucose uptake, the more insulin concentrations rise. This is why both insulin concentration and glucose are built into the minimal model equations. During the conduct of the FSIGT, both glucose and insulin are collected at 32 time points. Indeed the equation for S_I_ calculates the net rate of change of glucose over time at an insulin concentration above basal. Acute insulin response to glucose (AIRg) was determined by the area under the insulin curve between 0 and 10 min for the insulin concentration above basal. VAT and subcutaneous adipose tissue (SAT) were measured at L2-3 using a GE Hispeed Advantage CT/I scanner (Milwaukee, WI) and analyzed on a SUN workstation (MEDx image, Sensor System, Inc., Sterling, VA). Percent body fat was determined with a dual energy X-ray absorptiometry (DXA) scan (Hologic QDR4500A, Bedford, MA).

### Standard diet and test meal

The controlled diet began between 1 and 14 days after the IM-FSIGT. During the 7 day controlled dietary period, all meals were prepared in the metabolic kitchen at the NIH Clinical Center. On weekdays, participants reported in the morning to be weighed and eat breakfast. Lunch and dinner were provided in a cooler for consumption off-site. Compliance with picking up daily food packet and weekend food cooler was 100%. On day 8, the participants came to the Clinical Center at 7 AM after a 12 hour fast. The test meal was consumed. On Day 8 breakfast was provided with 30% of the energy consumed on Day 7 of the standard diet (21). The meal was an egg omelet with butter and cheddar cheese, plain bagel with cream cheese and orange juice (40% fat, 40% carbohydrate and 20% protein). Blood samples were obtained fasting and 2, 4 and 6 hours postprandially.

### Standard diet

As described previously, the Mifflin St. Jeor equation multiplied by a physical activity factor was used to estimate energy needs [21]. The physical activity factor was based on a dietician initiated interview and the National Research Council Dietary Reference Intake scale. An activity factor of 1.00-1.39 is for sedentary activity; 1.40-1.59 for sedentary activity plus 30–60 min of moderate activity; 1.60-1.89 for sedentary activity plus >60 min of daily moderate activity; and 1.90-2.50 for sedentary activity plus >60 min of daily moderate activity and >60 min of vigorous activity or >120 min of moderate activity. The macronutrient distribution of the meal was based on a typical American diet (33% fat, 52% carbohydrate, 15% protein).

### Ultracentrifugation

Whole plasma samples were separated into density factions by ultracentrifugation [21]. Four density fractions of plasma were collected: light VLDL (d < 1.006 g/mL, Svedberg units of flotation (Sf): 60 ∼ 400), dense VLDL (d < 1.006 g/mL, Sf: 20 ∼ 60), IDL (1.006 g/mL < d < 1.025 g/mL), LDL (1.025 g/mL < d < 1.063 g/mL) and HDL (d > 1.063 g/ml).

### ApoCIII measurement

Sandwich ELISA procedures using affinity-purified antibodies (Academy Biomedical Company Inc., Houston, TX) were performed to determine the apoCIII concentrations in whole plasma and lipoprotein fractions. All assays were completed in triplicate.

### ApoAI measurement

We measured the apoAI concentration of HDL with or without apoCIII concentration by ELISA. ELISA wells were coated with polyclonal anti-apoCIII which captured plasma lipoproteins that contain apoCIII. The apoAI concentration of the unbound fraction was measured by standard sandwich ELISA using polyclonal anti-apoAI for capture and detection. This step measured the apoAI concentration of HDL that does not contain apoCIII. The lipoproteins bound to the anti-apoCIII plate were dissociated with Tween-20, and the apoAI concentration was measured by the same sandwich ELISA. This step measured the apoAI concentration of HDL that contains apoCIII. All assays were completed in triplicate.

### Statistical analyses

Differences in demographic and metabolic characteristics between African-Americans and whites were determined with two-tailed unpaired t-test. *P*-values ≤0.05 were considered significant. Generalized least squares random effects models (REM) were used to examine race differences in the lipid response to the meal using measurements from samples collected both fasting and 2, 4 and 6 hours postprandially. All models included race and time as categorical variables. Because S_I_ and VAT differ between African-Americans and whites, and they also affect lipoprotein metabolism, we performed a series of multiple variable models to gain understanding of how S_I_ and VAT may influence the racial differences in apoCIII containing lipoproteins. Models were built by adding S_I_ and VAT singly and together, and their effect on the coefficient for race examined. Analyses were performed with STATA, v12.0 (College Station, Texas).

## Results

### Demographic and metabolic characteristics of the participants

Briefly, thirty-two non-diabetic women (14 African-American and 18 whites) were enrolled. Age and BMI ranges were 22 to 59 years and 20.6 to 45.9 kg/m^2^, respectively. By design, the women were similar in age (Table 1). Mean BMI was 34.3 kg/m^2^ in the African – Americans compared to 30.1 kg/m^2^ in the whites (*P* = 0.06). The two groups had similar percent body fat and waist circumference. However, thigh circumference tended to be higher in the African-Americans (*P* = 0.01). The African-American women had less VAT than the whites (*P* = 0.03). Subcutaneous abdominal adipose tissue did not differ by race (*P* = 0.37). The African-Americans were more insulin-resistant (lower S_I_, *P* = 0.01) and hyperinsulinemic (higher AIRg, *P* <0.001) than whites.

**Table 1:** **Demographic and metabolic characteristics in participants** ^**1**^

Variable	African-Americans (n = 14)	Whites (n = 18)	***P-***Value ^2^
Age (y)	39 ± 12	42 ± 11	0.45
BMI	34.3 ± 7.4	30.1 ± 4.9	0.06
Percent body fat (%)	39.7 ± 9.1	38.8 ± 7.8	0.8
Waist circumference (cm)	101.0 ± 17	100.6 ± 15	0.95
Thigh circumference (cm)	66 ± 11	57 ± 7	0.01
VAT (cm^2^) ^3^	109 ± 74	114 ± 66	0.03
SAT (cm^2^) ^3^	341 ± 187	271 ± 139	0.37
S_I_ (mU/L^-1^.min^-1^)	3.0 ± 1.5	5.3 ± 2.5	0.01
AIRg (mU.I^-1^.min)	760 ± 437	215 ± 176	<0.001
Family history of diabetes	43% (6/14)	44% (8/18)	0.93
No college degree	43% (6/14)	6% (1/18)	0.01
Graduate school	36% (5/14)	56% (10/18)	0.28
Smokers	2/14	5/18	0.38

^1^Data presented as mean ± SD.

^2^Comparisons by unpaired t-tests.

^3^ *P*-value adjusted for BMI with regression analysis.

### Fasting lipids and apolipoproteins

Fasting lipids and apolipoproteins did not differ significantly by race (Table 2).

**Table 2:** **Fasting lipids and apolipoproteins** ^**1**^

Variable	African-Americans (n = 14)	Whites (n = 18)	***P***-Value ^2^
Cholesterol (mg/dL)	185 ± 41	172 ± 35	0.20
TG (mg/dL)	79 ± 45	98 ± 50	0.15
HDL cholestrol (mg/dL)	56 ± 14	49 ± 11	0.10
LDL cholesterol (mg/dL)	113 ± 34	102 ± 29	0.23
ApoAI (mg/dL)	175 ± 32	167 ± 30	0.52
Apo AI of HDL with ApoC-III (mg/dL)	13.2 ± 3.0	12.7 ± 2.6	0.57
Apo AI of HDL without ApoC-III (mg/dL)	162 ± 30	154 ± 28	0.52
ApoCIII (mg/dL)	10.4 ± 3.7	9.6 ± 3.9	0.55
HDL-ApoCIII (mg/dL)	7.0 ± 2.9	5.7 ± 3.1	0.23
Light VLDL-ApoCIII (mg/dL)	1.5 ± 1.4	1.7 ± 1.1	0.60
Dense VLDL-ApoCIII (mg/dL)	0.61 ± 0.55	0.93 ± 0.51	0.10
IDL-ApoCIII (mg/dL)	0.28 ± 0.29	0.32 ± 0.28	0.68
LDL-ApoCIII (mg/dL)	1.0 ± 0.6	1.0 ± 0.5	0.68

^1^Data presented as mean ± SD.

^2^Comparisons by unpaired t-tests.

### ApoAI concentration in HDL subfractions during the test meal

**Figure 1:**
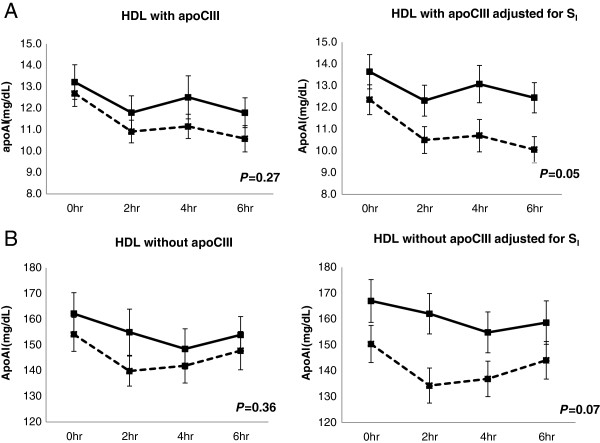
**ApoAI concentrations of HDL with or without apoCIII at baseline, 2, 4 and 6 hours postprandially.** The rows present the apoAI concentration in HDL with apoCIII **(A)** and HDL without apoCIII **(B)**. Panels present unadjusted (left panel) and adjusted for S_I_ (right panel). Data is from random effect multiple models constructed to determine the effect of race on the apoAI concentration in each HDL subfraction. The *P*-value for the effect of race is presented in each diagram. African-American women: solid lines; white women: dashed lines.

The apoAI concentrations of HDL with or without apoCIII from baseline through 6 hours during the test meal were not significantly different by race (Figure 1, left panels). However, after controlling for S_I_, African-American women had significantly higher apoAI level in HDL with apoCIII than whites (mean ± SD: 12.9 ± 2.8 and 10.9 ± 2.9 mg/dL, respectively, *P* = 0.05) (Figure 1A, right panel). The difference between the groups in apoAI in HDL with apoCIII increased over the 6-hour postprandial study. African-American women also tended to have higher apoAI level in HDL without apoCIII than whites (Figure 1B, right panel).

### Determinants of apoAI concentration in each HDL subfraction

Because S_I_ and VAT were significantly lower in the African-American group, the effects of S_I_ and VAT on the difference by race in the HDL subfractions were studied in multivariable models. The apoAI concentration of HDL with apoCIII was significantly directly associated with S_I_. The regression coefficient on HDL with apoCIII of race doubled after adjustment for S_I_ (Table 3, Model A: β-coef =1.00, Model B: β-coef = 1.96). African-American women also tended to have higher apoAI level in HDL without apoCIII after controlling for S_I_; which itself was associated with HDL without apoCIII (Table 3, Model B). VAT was not significantly related to HDL with apoCIII or HDL without apoCIII (Table 3, Model C). When race, S_I_ and VAT were included as independent variables (Table 3 Model D), apoAI level in HDL with apoCIII in African-American women was significantly higher than in whites. Both race and S_I_ were significant determinants of apoAI concentration in HDL with apoCIII; overall R^2^ = 14% (both *P* = 0.04). In this combined model, none of these variables made a significant contribution to apoAI content of HDL without apoCIII (Table 3, Model D).

**Table 3:** **Random effects model to determine influence of race** ^**1**^ **, S**_**I**_ **and VAT on apoA-I level**

ApoAI of HDL with apoCIII					ApoAI of HDL without apoCIII			
Model	Variables	β-coef	SE	***P***-val	Variables	β-coef	SE	***P***-val
A		R^2^ = 3%				R^2^ = 2%		
	Race	1.00	0.90	0.27	Race	8.96	9.87	0.36
		R^2^ = 13%				R^2^ = 12%		
B	Race	1.96	0.98	0.05	Race	19.23	10.75	0.07
	S_I_	0.42	0.21	0.04	S_I_	4.47	2.25	0.05
		R^2^ = 3%				R^2^ = 7%		
C	Race	0.98	0.92	0.28	Race	8.49	9.79	0.39
	VAT	-0.002	0.007	0.73	VAT	-0.09	0.07	0.22
		R^2^ = 14%				R^2^ = 13%		
D	Race	2.16	1.03	0.04	Race	18.01	11.39	0.11
	S_I_	0.50	0.23	0.04	S_I_	4.00	2.60	0.12
	VAT	0.005	0.007	0.50	VAT	-0.03	0.08	0.71

^1^Whites are the referent group.

### ApoCIII concentration in HDL and apoB lipoproteins during the test meal

**Figure 2:**
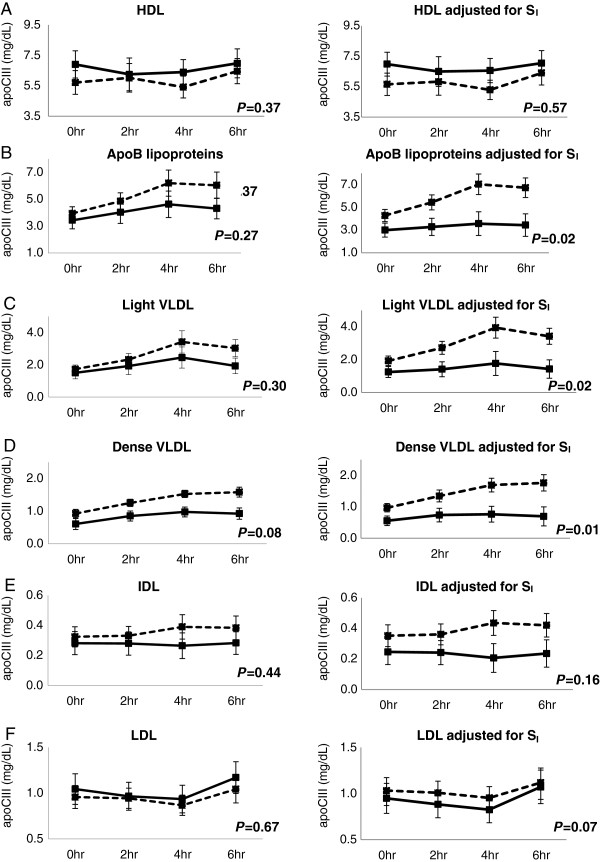
**ApoCIII concentrations in HDL and apoB lipoproteins at baseline, 2, 4 and 6 hours postprandially.** The rows present the apoCIII concentration in HDL **(A)**, total apoB lipoproteins **(B)**, light VLDL **(C)**, dense VLDL **(D)**, IDL **(E)** and LDL **(F)**. Columns present unadjusted (left panel) and adjusted for S_I_ (right panel). Data are from random effect multiple models constructed to determine the effect of race on the apoCIII concentration in each lipoprotein. The *P*-value for the effect of race is presented in each diagram. African-American women: solid lines; white women: dashed lines.

The apoCIII concentrations of HDL, total apoB lipoproteins, light VLDL, dense VLDL, IDL and LDL during the test meal were not significantly different by race (Figure 2, left panels). However, after controlling for S_I_, African-American women have significantly lower apoCIII level in total apoB lipoproteins, light VLDL and dense VLDL than whites, *P* = 0.02, 0.02 and 0.01 respectively. (Figure 2B, C and D, right panels). The differences between the groups in apoCIII in total apoB lipoproteins, light VLDL, dense VLDL and IDL increased during the 6-hour postprandial study. There were no significant differences in apoCIII levels in HDL, IDL or LDL by race even after controlling for S_I_ (Figure 2A, E and F, right panels).

### Determinants of apoCIII levels in HDL and apoB lipoproteins

The influence of S_I_ and VAT on the difference between the groups in the apoCIII concentrations in HDL and apoB lipoproteins was studied in multivariable models. Controlling for S_I_, African-American women had lower apoCIII in total apoB lipoproteins, light VLDL and dense VLDL than whites (Table 4, model B). S_I_ was inversely related to apoCIII level in light VLDL and total apoB lipoproteins. Neither race nor S_I_ was a determinant of apoCIII concentration in HDL, IDL and LDL.

**Table 4:** **Random effects model to determine influence of race** ^**1**^ **, S**_**I**_ **and VAT on apoCIII level**

	ApoCIII – HDL				ApoCIII - apoB lipoproteins					ApoCIII - Light VLDL		
Model	Variables	β-coef	SE	***P***-val	Variables	β-coef	SE	***P***-val	Variables	β-coef	SE	***P***-val
A		R^2^ = 2 %				R^2^ = 3 %				R^2^ = 3 %		
	Race	0.97	1.09	0.37	Race	-1.16	1.06	0.27	Race	-0.68	0.65	0.30
		R^2^ = 3 %				R^2^ = 18 %				R^2^ = 17 %		
B	Race	0.73	1.27	0.57	Race	-2.55	1.11	0.02	Race	-1.54	0.68	0.02
	S_I_	-0.11	0.27	0.69	S_I_	-0.60	0.23	0.01	S_I_	-0.38	0.14	0.01
		R^2^ = 2 %				R^2^ = 25 %				R^2^ = 23 %		
C	Race	0.98	1.11	0.38	Race	-1.04	0.91	0.25	Race	-0.60	0.56	0.28
	VAT	0.001	0.01	0.86	VAT	0.02	0.01	0.001	VAT	0.01	0.004	0.001
		R^2^ = 3 %				R^2^ = 29 %				R^2^ = 27 %		
D	Race	0.72	1.35	0.59	Race	-1.81	1.07	0.09	Race	-1.10	0.66	0.09
	S_I_	-0.11	0.31	0.72	S_I_	-0.32	0.24	0.19	S_I_	-0.21	0.15	0.16
	VAT	-0.0001	0.01	0.99	VAT	0.02	0.01	0.01	VAT	0.01	0.005	0.02
	**ApoCIII - dense VLDL**				**ApoCIII – IDL**				**ApoCIII – LDL**			
**Model**	**Variables**	**β-coef**	**SE**	***P*** **-val**	**Variables**	**β-coef**	**SE**	***P*** **-val**	**Variables**	**β-coef**	**SE**	***P*** **-val**
A		R^2^ = 7 %				R^2^ = 2 %				R^2^ = 0.5 %		
	Race	-0.48	0.27	0.08	Race	-0.08	0.10	0.44	Race	0.08	0.18	0.67
		R^2^ = 15 %				R^2^ = 8 %				R^2^ = 8 %		
B	Race	-0.75	0.3	0.01	Race	-0.16	0.12	0.17	Race	-0.10	0.20	0.63
	S_I_	-0.12	0.06	0.06	S_I_	-0.03	0.02	0.16	S_I_	-0.07	0.04	0.07
		R^2^ = 21 %				R^2^ = 16 %				R^2^ = 10 %		
C	Race	-0.46	0.25	0.07	Race	-0.07	0.10	0.47	Race	0.09	0.17	0.60
	VAT	0.005	0.002	0.01	VAT	0.002	0.001	0.02	VAT	0.003	0.001	0.04
		R^2^ = 23 %				R^2^ = 16 %				R^2^ = 12 %		
D	Race	-0.59	0.3	0.05	Race	-0.10	0.12	0.40	Race	-0.02	0.20	0.93
	S_I_	-0.05	0.07	0.43	S_I_	-0.01	0.03	0.26	S_I_	-0.05	0.05	0.32
	VAT	0.004	0.002	0.05	VAT	0.001	0.001	0.08	VAT	0.002	0.001	0.18

^1^Whites are the referent group.

With race and VAT included as independent variables, VAT was a significant determinant of apoCIII concentration in total apoB lipoproteins, light VLDL, dense VLDL, IDL and LDL (all *P* ≤0.04), but did not influence the relation between race and apoCIII (Table 4, model C). Importantly, the overall R^2^ for total apoB lipoproteins, light VLDL, dense VLDL and IDL model C were higher than for the preceding model in which the two independent variables were race and S_I_ indicating the importance of VAT in determining levels of apoCIII in total and specific apoB lipoproteins. After adding both S_I_ and VAT to the same model, VAT appeared to have a dominating influence over S_I_ on apoCIII in total apoB lipoproteins, light VLDL, dense VLDL and IDL (Table 4, model D).

## Discussion

We report for the first time that African-American compared to white women have a higher level of apoAI in HDL with apoCIII, an HDL subspecies that is associated with increased risk of CHD [15], after controlling for differences in insulin sensitivity. African-American women also tended to have higher levels of apoAI in HDL without apoCIII, which has a protective association with CHD expected for HDL, although not significantly in the fully adjusted model that adjusted for differences in insulin sensitivity and VAT. The African-American group also tended to have a lower apoCIII level in apoB containing lipoproteins than whites after controlling for insulin sensitivity and VAT. These differences between the groups in apoAI with apoCIII, and in apoCIII in VLDL and IDL increased during the postprandial period, indicating the importance of studying lipoproteins in response to food intake. Since higher levels of HDL without apoCIII and lower levels of apoliprotein CIII predict lower rates of CHD, the results would suggest lower rather than higher prevalence of CHD in African-American women, unless the higher level of HDL with apoCIII is somehow a dominating influence. We do not know how these lipoprotein-related risk factors predict CHD in African-American populations.

**Figure 3:**
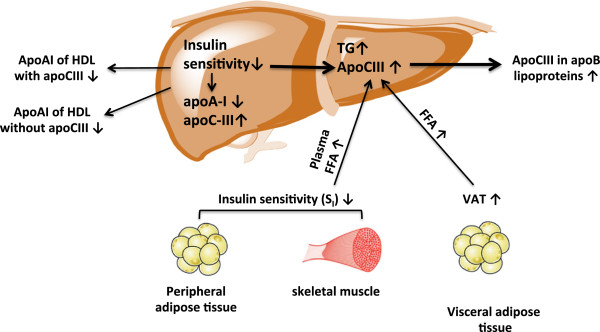
**Metabolic pathways suggested by the results of this study.** Increased visceral adipose tissue increases flux of FFA to the liver. The liver responds by increasing the secretion of apoC-III in apoB lipoproteins. Lower total body insulin sensitivity including in skeletal muscle and peripheral adipose tissue increases plasma FFA, which increases secretion of apoC-III in apoB lipoproteins. Lower insulin sensitivity reduces apoAI level in HDL with apoCIII, and in HDL without apoCIII. African-American race is associated with increased apoAI of HDL with apoCIII, and decreased apoCIII in apoB lipoproteins, independent of differences in insulin sensitivity and VAT.

We found a complex interrelation among insulin sensitivity, VAT, and racial group, as determinants of apoAI lipoprotein concentrations and apoCIII concentrations in VLDL and IDL (Figure 3). Insulin sensitivity, measured directly by IM-FSIGT, was associated with higher apoAI concentration of both HDL subspecies with and without apoCIII. Insulin sensitivity was also associated with lower levels of apoCIII in total apoB lipoproteins and in VLDL. Because African-Americans have less insulin sensitivity, apoAI would be expected to be lower and apoCIII higher than whites, which is not true. In fact, at any level of insulin sensitivity, African-Americans have higher apoAI with apoCIII and lower apoCIII in apoB lipoproteins. Thus, we hypothesize that there must be strong positive metabolic mechanisms in African-Americans to counteract the effect on apoAI with or without apoCIII, and apoCIII in apoB lipoproteins of low insulin sensitivity.

Low mass of visceral adipose tissue in African-Americans is a candidate mechanism. VAT indeed was associated with higher levels of apoCIII in total apoB lipoproteins, VLDL, IDL and LDL. However, VAT was not associated with apoAI in HDL with or without apoCIII. Thus, the low VAT in African-Americans could counterbalance adverse metabolic effects of low insulin sensitivity at least on apoCIII in apoB lipoproteins. VAT does appear to have a dominating influence over S_I_ on apoCIII in apoB lipoproteins as shown by models that include both S_I_ and VAT. Still, VAT and insulin sensitivity did not explain the full difference between the groups in apoCIII in light and dense VLDL and in apoB lipoproteins and in apoAI with apoCIII.

Insulin regulates apoAI and apoCIII transcription [27, 28]. In vitro studies showed insulin upregulated apoAI transcription in hepatocytes through an insulin-responsive core element (IRCE). The IRCE contains a Sp1 binding site, and insulin treatment is associated with serine/threonine phosphorylation of Sp1 resulting in increased Sp1 binding to IRCE [29, 30]. Altomonte et al. reported that insulin inhibited apoCIII gene expression and synthesis. Forkhead box O1 (Foxo 1), a nuclear transcription factor, is associated with the inhibitory effect of insulin on apoCIII expression [31]. In our study, insulin sensitivity is positively associated with apoAI in HDL with apoCIII or without apoCIII but inversely with apoCIII in apoB lipoproteins. All this evidence suggests that insulin may induce an opposing effect on apoAI level and apoCIII level through the transcriptional regulation of these two apolipoproteins.

In lean individuals with normal insulin sensitivity and people with insulin resistance, African-Americans have significantly lower apoCIII levels than whites [19, 20]. In states of insulin resistance, an inhibitory role of insulin on apoCIII expression may be lost [32]. Florez et al. reported that the increment in apoCIII level associated with diabetes status and higher degree of insulin resistance was evident in Hispanics and white non-Hispanics but not in African-Americans [20]. Thus, at a similar level of insulin resistance, African-Americans have lower apoCIII than whites.

Kinetics studies in human subjects have shown that increased production rate of apoCIII rather than decreased apoCIII fractional catabolic rate is a determinant of the elevated plasma apoCIII levels that characterize patients with hypertriglyceridemia and features of insulin resistance [33], consistent with the effect of insulin resistance on the apoCIII promoter. In fact, evidence suggests that African-Americans may have lower apoCIII production rate than whites [20, 33]. There are several factors that may contribute to the difference of apoCIII production rate in addition to insulin between African-American and whites. In vitro, activation of PPAR alpha is associated with reduced apoCIII gene expression. Shin et al. reported that two minor allele frequencies, L162V and rs4253728 which were associated with the increases in plasma levels of apoCIII, were significantly lower in African Americans compared with whites [19]. Plasma free fatty acids stimulate apoCIII production in humans [34]. Miller et al. reported that obese African-American women with T2DM have lower postabsorptive plasma FFA that whites [35]. All these evidence suggest that genetic variants in the apoCIII promoter region and lower postprandial plasma FFA in African-Americans may induce lower apoCIII level than whites.

### Study limitations

The two major limitations of the study are the lack of kinetics data on lipoproteins to explore mechanisms and our sample size of 32 which may have rendered the study underpowered to detect differences between the groups. The numerous borderline significant p-values may reflect the relatively small sample.

### Study strengths

A major strength of study is how similar the African-American and white women were in age, BMI, family history of diabetes and socioeconomic factors. An additional strength is that the women were provided with a diet with the same daily distribution of nutrients for one week prior to the postprandial study. Furthermore, we directly measured S_I_ using the Minimal Model.

## Conclusion

African-American women have higher apoAI in HDL with apoCIII and lower apoCIII in apoB lipoprotein postprandially. The differences between the groups in apoAI in HDL with apoCIII, and in apoCIII in VLDL and IDL increased during the postprandial period, indicating the importance of studying lipoproteins in response to food intake. We found a complex interrelation among insulin sensitivity, VAT and racial group, as determinants of apoAI lipoprotein concentrations and apoCIII concentrations in VLDL and IDL. The differences in HDL with apoCIII level and apoCIII in apoB lipoproteins level between races are obscured by lower insulin sensitivity, because insulin sensitivity is associated with higher levels of HDL with apoCIII and lower levels of apoCIII in apoB lipoproteins. In contrast to SI, VAT has a strong association with apoCIII in apoB lipoproteins but not with apoAI with apoCIII level. VAT and insulin sensitivity did not explain the full difference between the groups in apoCIII in light and dense VLDL and in apoB lipoproteins and in apoAI with apoCIII. However, our results suggest that insulin sensitivity and VAT contribute to the differences in lipoprotein levels, which are associated with CHD risk, between African-American women and white women.
